# Retrograde Solubility of Methylammonium Lead Iodide in γ-Butyrolactone Does Not Enhance the Uniformity of Continuously Coated Films

**DOI:** 10.1021/acs.langmuir.3c03979

**Published:** 2024-04-18

**Authors:** Maimur Hossain, Jesse L. Starger, Jesse J. Efymow, Ryan F. Barrett, Jacob S. Bolduc, Nicolas J. Alvarez, Richard A. Cairncross, Aaron T. Fafarman, Jason B. Baxter

**Affiliations:** † Department of Chemical and Biological Engineering, Drexel University, Philadelphia, Pennsylvania 19104, United States; ‡ Department of Materials Science and Engineering, Drexel University, Philadelphia, Pennsylvania 19104, United States

## Abstract

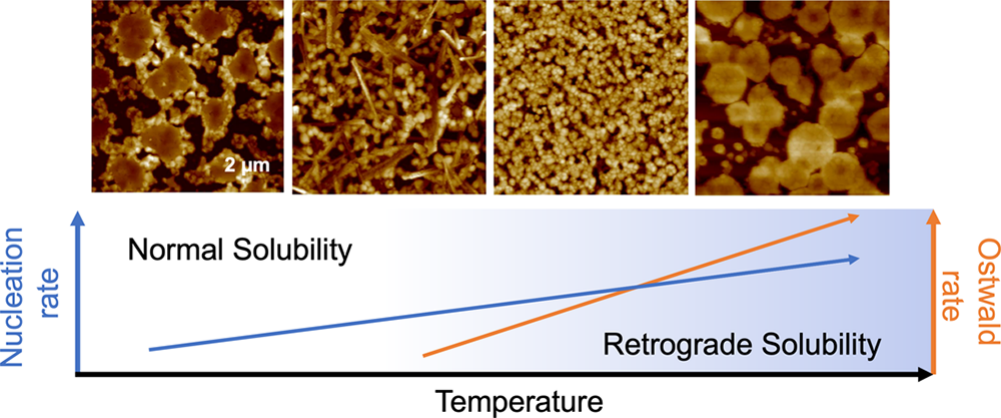

Halide perovskite thin films can be the centerpiece of high-performance solar cells, light-emitting diodes, and other optoelectronic devices if the films are of high uniformity and relatively free of pinholes and other defects. A common strategy to form dense films from solution has been to generate a high density of nuclei by rapidly increasing supersaturation, for example, by timely application of an antisolvent or forced convection. In this work, we examine the role of retrograde solubility, wherein solubility decreases with increasing temperature, as a means of increasing the nucleation density and film coverage of slot-die-coated methylammonium lead iodide (MAPbI_3_) from γ-butyrolactone (GBL) solution. Coverage was investigated as a function of the substrate temperature and the presence and temperature of an air knife. Results were considered within the framework of the dimensionless modified Biot number, which quantifies the interplay between evaporation and horizontal diffusion. Moderate temperatures and a heated air knife improved film coverage and morphology by enhanced nucleation up to ∼80 °C. However, despite the dense nucleation enabled by retrograde solubility, slow evaporation as a result of the low vapor pressure of GBL, combined with Ostwald ripening at high temperatures, prevented the deposition of void-free, device-quality films. This work has provided a more detailed understanding of the interplay between perovskite processing, solvent parameters, and film morphology and ultimately indicates the obstacles to forming dense, uniform films from solvents with high boiling points even in the presence of rapid nucleation.

## Introduction

Halide perovskite materials with the formula ABX_3_, where A = CH_3_NH_3_^+^/HC(NH_2_)_2_^+^/Cs^+^, B = Pb/Sn, and X = I, Br, or Cl, are under intense research and development for photovoltaic and other optoelectronic applications.^1−6^ In addition to their remarkable optoelectronic qualities, their facile and inexpensive solution processability may give them techno-economic advantages for commercialization.^7^ This processability has been demonstrated from several solvents.^8−13^ Some are highly represented in the literature on thin-film synthesis, such as *N*,*N*-dimethylformamide (DMF) and dimethyl sulfoxide (DMSO), while others, such as γ-butyrolactone (GBL), are widely used for single-crystal growth. When considering frequently used co-solvents, the list expands considerably.

In the context of perovskite photovoltaics, the objective is to deposit a uniform, pinhole-free polycrystalline thin film with crystal grains that are large in comparison to the film thickness. While much progress has been made toward this objective using the approach of spin casting,^8,9,14^ industrial implementation requires a large-area, continuous deposition process, such as blade or slot-die coating,^15,16^ for which it is even more challenging to achieve the desired outcome. The crux of this challenge is the interplay between solvation, drying, and crystallization. Fortuitously, the desired crystal grain size can be achieved in post-deposition annealing, which provides controllable grain coarsening.^17,18^ The remaining primary objective, therefore, is to minimize the existence of voids or pinholes in the as-deposited, dry films. A common approach to this singular objective is to increase the rate of supersaturation in the wet film, thereby causing a proliferation of small nuclei to densely cover the substrate, using techniques including application of an antisolvent during drying (“solvent drip”^19,20^ or “solvent–solvent extraction”^21,22^) or accelerating solvent evaporation by adding forced convection (“vacuum flash”^23^ or “gas quenching”^24,25^) or increasing the coating temperature (e.g., hot casting).

The present study asks whether the phenomenon of retrograde solubility, wherein solubility decreases as the temperature increases beyond a certain threshold, such as is known for certain combinations of perovskite precursors and solvents,^26^ can similarly enhance nucleation density to the benefit of film coverage. The basis for positing such an effect comes from classical nucleation theory, which predicts that the nucleation rate goes through a maximum value with respect to the temperature, with the negative slope predicated on the driving force for crystallization (degree of supersaturation) decreasing with increasing temperature as a result of greater solubility.^26^ Retrograde solubility upends this presumption because the driving force for nucleation, namely, the degree of supersaturation, instead *increases* with increasing temperature, thereby acting in concert with both the increase in thermal activation and faster diffusion of the solute to any incipient nucleus. Synergistically, the drying rate will also increase with temperature, further accelerating supersaturation. This analysis suggests that the nucleation rate could simply increase monotonically with temperature for retrograde solubility. However, two attendant effects of enhanced diffusivity at elevated temperature could work against increased nucleation, to the detriment of film uniformity: fast diffusion of all nearby solute to the nearest nucleus before the wet film has dried enough to vertically constrain growth, leading to tall crystals and voids in between, or accelerated Ostwald ripening, again growing large crystals at the expense of many small nuclei.

**Figure 1:**
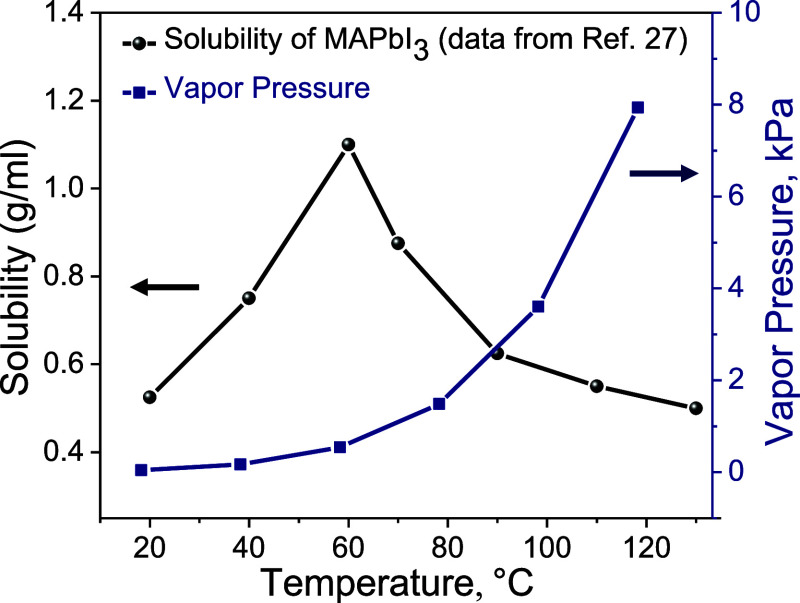
Solubility of MAPbI_3_ in GBL and vapor pressure as a function of temperature. Solubility data from ref (27) show retrograde behavior above 60 °C. The vapor pressure was calculated from Antoine parameters from ref (28).

The solubility of methylammonium lead iodide (MAPbI_3_) in GBL increases with increasing temperature (normal solubility) up to 60 °C and then decreases at higher temperatures (retrograde solubility),^27^ as shown in Figure 1. The Antoine equation was used to estimate the temperature-dependent vapor pressure for GBL using reported values of Antoine parameters.^28^ Higher temperatures increase the vapor pressure of GBL (Figure 1) and, consequently, the drying rate, thus synergistically facilitating rapid nucleation. Retrograde solubility has been exploited for growth of large single crystals from GBL,^27^ but to our knowledge, there are no reports of GBL as a pure solvent being used above its retrograde solubility point for perovskite thin-film fabrication. Herein, we test whether retrograde solubility might be utilized as a tool to increase the nucleation rate in a way that is more amenable to continuous coating techniques than, for example, antisolvent methods. Because elevating the temperature will also increase diffusion, with the risk of unconstrained crystal growth, the effect of an air knife to increase the convective removal of solvent vapors is tested. Under convective flow, drying can be accelerated independently of other effects, and we tested whether this sufficiently reduces the time available for undesired long-range diffusion. The primary outcome measured is the coverage of the film and the occurrence of pinholes as a function of temperature and convective flow.

We utilize the concept of the dimensionless modified Biot number (Bi*), which compares the evaporation velocity to the horizontal diffusion velocity, to provide a theoretical framework to understand how film quality depends upon the interplay of nucleation, growth, and drying.^29^Bi* estimated from morphological data herein and known solvent properties indicates significant impediments toward a wider use of pure GBL as a processing solvent for perovskite coatings and points to the value of pursuing retrograde solubility in solvents with significantly faster evaporation rates.

## Experimental Methods

### Materials

PbI_2_ (99.8%) and methylammonium iodide were sourced from TCI. GBL was purchased from Sigma-Aldrich. All chemicals were used as received. A concentration of 0.4 M (0.25 mg/mL) MAPbI_3_ was used.

### Instruments

MAPbI_3_ films were coated using a slot-die plate coater (Ossila L2005A1, U.K.) with a temperature-controlled stage. Optical microscope images were collected with an Olympus CH30. Rigaku SmartLab was utilized for X-ray diffraction (XRD) analysis. Atomic force microscopy (AFM) analysis on perovskite films was studied using a Bruker Icon atomic force microscope.

### Film Processing

Glass slides (25 × 75 mm) were cleaned sequentially using Hellmanex detergent solution, deionized (DI) water, acetone, and isopropanol in an ultrasonic bath for 15 min each, then dried, and treated with ultraviolet (UV) ozone. Perovskite films were slot-die-coated under ambient conditions over the full 25 mm width of the substrate. Typical coating conditions used an ink flow rate of 6.8 μL/s, a coating speed of 30 mm/s, and a coating gap of 200 μm. All films were coated onto substrates that were preheated to the indicated stage temperature (25–120 °C) with no further annealing after coating; hence, it was possible to observe thermally unstable intermediates for the films cast at room temperature.

In a subset of coating conditions, as indicated later in the text, an air knife was used to accelerate drying. The air knife was oriented in cross-flow as a result of restrictions with the geometry and 100 mm limit of travel of the plate coater (Figure S1 of the Supporting Information). This configuration was found to have no negative impact on the uniformity within the central region of the film (the region of interest in subsequent characterizations). A total of 1 standard cubic f of the Supporting Information). This configuration was found to have no negative impact on the uniformity within the central region of the film (the region of interest in subsequent characterizations). One standard cubic foot per minute (SCFM) was chosen as the highest practicable flow rate for this air knife geometry; higher flow rates were found to disturb the wet film during coating. Hot-wire anemometer readings were used to confirm that linear velocities were greater than 2 m/s near the substrate, which was demonstrated by Ternes et al. to be the minimum velocity needed to maintain acceptable root-mean-square (RMS) surface roughness for films deposited from DMF at temperatures up to 85 °C.^34^ In some cases, the air was preheated to the substrate temperature as noted in the Results and Discussion.

## Results and Discussion

The temperature plays many roles in both the kinetic and thermodynamic aspects of crystallization. In addition to influencing nucleation and drying rates, the temperature also impacts the preferred crystal composition, phase, and morphology that forms first when drying solutions of MAPbI_3_ in GBL. For example, Fateev et al. have demonstrated that the crystal that forms during drying at room temperature is a solvent-adduct phase, such as MA_8_(GBL)_*x*_Pb_18_I_44_ or MA_2_(GBL)_2_Pb_3_I_8_, that frequently exhibits a needle-like morphology.^30^ As shown in Figure S2 of the Supporting Information, the starting morphology is preserved even when the temperature is subsequently increased, and the solvent-adduct phase converts to pure crystalline MAPbI_3_.

**Figure 2:**
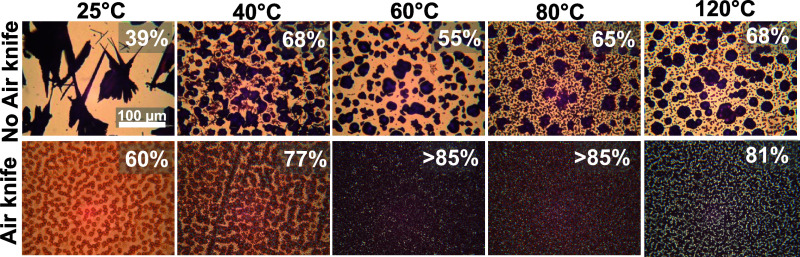
Optical microscope images of perovskite films coated without (top) and with (bottom) a room-temperature air knife at different substrate temperatures. The scale bar applies to all images. Numbers in the upper right of each panel indicate the surface coverage.

We began to test the hypothesis that retrograde solubility and drying rate can be used to control the film structure by slot-die coating with the stage temperature from 25 to 120 °C, with or without an air knife. To prevent crystallization in the ink at room temperature and to enable time for the wet film to reach the desired temperature before crystallization, we used a concentration of 0.4 M (0.25 g/mL) MAPbI_3_ solution in GBL, which is below saturation according to Figure 1 (details can be found in the Experimental Methods). Figure 2 shows optical micrographs taken of the dry, as-deposited films subject to different deposition and drying conditions, with estimation of coverage (upper right inset) performed automatically using ImageJ software. For films processed at 25 °C, a 100 μm-scale needle or star-shaped microstructure was apparent along with very poor coverage (∼39%), as seen in the upper left panel. This film morphology matches that of the initially formed solvent-adduct microcrystalline phases described above;^30^ the retention of such incipient microcrystalline morphology in the final film is a phenomenon also observed in MAPbI_3_ deposited from DMF under conditions that similarly allow needle-like solvent-adduct microcrystals to form.^31^ In the film processed at 40 °C, a few needle-shaped microstructures were dispersed among the dominant circularly shaped MAPbI_3_ microstructures. As the processing temperature increased beyond 60 °C, rapid formation of black microstructures is evident by eye even before the film drying is complete. Under the microscope, the needle-shaped structures were no longer observed, and circular or volcano-shaped structures were evident in AFM images (Figure S3 of the Supporting Information). However, regardless of the processing temperature, the coverage remains poor (55–68%); large, sparse microstructures with heights of several micrometers are present at the expense of a uniform and dense thin film. This result suggests that the nucleation density, shape, and size of the microstructure can be modulated by the temperature, but the increase in the processing temperature alone does not improve the coverage sufficiently for device applications.

To significantly enhance the evaporation rate beyond heating alone (while also further increasing the nucleation density), an air knife was utilized along with the temperature variation of the slot-die stage (bottom row in Figure 2). The convective transport at the drying film surface is governed by the air flow rate, distance between the air knife and film, outlet width, and flow angle. These same parameters govern whether the convection is forceful enough to disrupt the wet film, negatively impacting the uniformity. Within the geometry dictated by other constraints in our system, an air flow rate of 1 SCFM was found to be effective in accelerating evaporation without redistributing the wet coating.

Use of an air knife significantly improved the overall coverage (80–85%). AFM images of films show that the tallest crystals are significantly shorter with an air knife than without (<300 nm for 80 °C and below), indicating a reduction in unconfined crystal growth with faster drying (Figures S3 and S4 of the Supporting Information). The processing temperatures of 60 and 80 °C with an air knife produced films with relatively close packing of smaller microstructures. However, as the processing temperature increased beyond 80 °C, the microstructure size increased significantly and bigger voids appeared in the films (Figure S4 of the Supporting Information). Under no conditions was the coverage satisfactory for photovoltaic devices.

**Figure 3:**
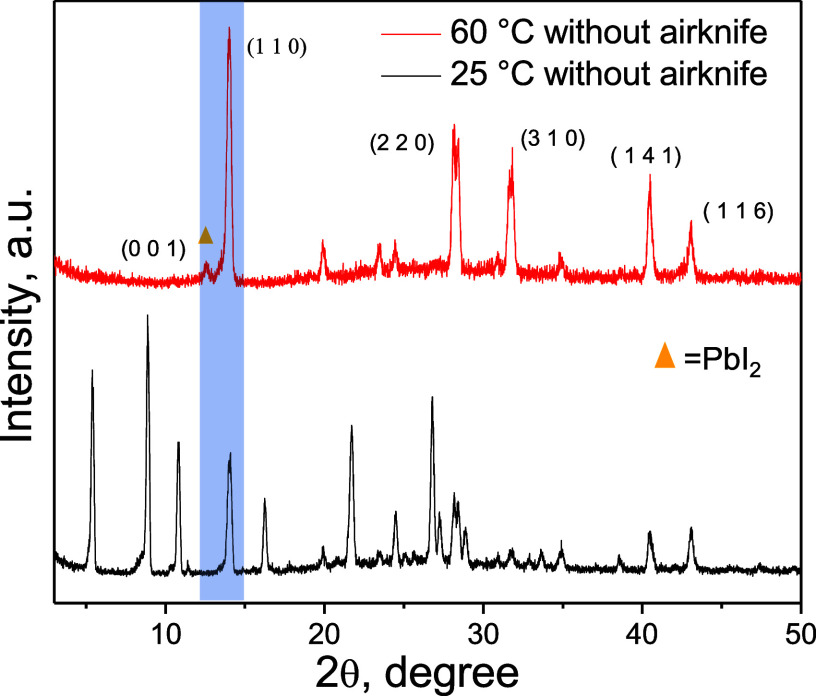
XRD patterns of films processed at 25 and 60 °C without an air knife. See Figure S5 of the Supporting Information for the XRD results of other films.

To confirm the formation and purity of the perovskite phase, XRD patterns of the thin films processed with variation of the stage temperature and without and with an air knife were analyzed (Figure 3 and Figure S5 of the Supporting Information). All of the films, except for the film deposited at 25 °C without an air knife, exhibited intense peaks at 14.1°, 28.4°, 31.8°, 40.5°, and 43.0° that are assigned to (110), (220), (310), (141), and (116) perovskite planes, respectively. The film deposited at 25 °C without an air knife had three peaks with 2θ less than 14°, which can be attributed to perovskite–solvent complexes,^30^ consistent with the morphological pattern of this film. Although there was a small peak at 2θ = 8° in the film processed at room temperature with an air knife, the overall pattern was otherwise like those of other films with fast drying.

**Figure 4:**
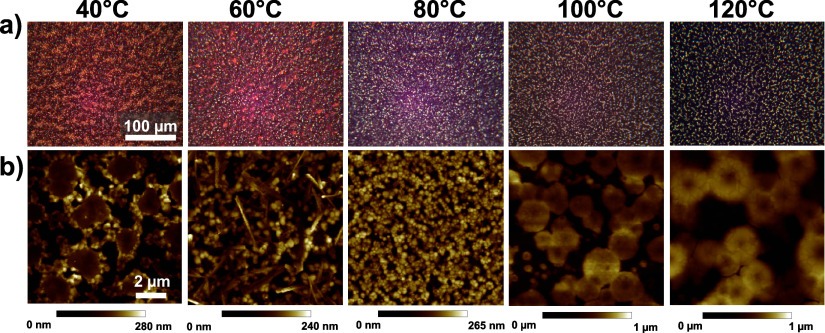
(a) Optical microscope images and (b) AFM images of perovskite films coated with a hot air knife at different temperatures. Scale bars apply to all images in the row.

The use of a room-temperature air knife in the experiments above resulted in a decrease of the effective temperature of the stage by ∼5–6 °C as a result of forced convection. To overcome this convective cooling and access even faster drying, the air supplied to the air knife was preheated to match the temperature of the stage. Figure 4 presents the microscope and AFM images of thin films coated with a hot air knife at different stage temperatures, while XRD in Figure S6 of the Supporting Information confirms the perovskite structure. The coverage and uniformity of domains significantly improved as temperatures increased up to 80 °C, which yielded the best condition for MAPbI_3_ coating from GBL solution, recapitulating the trend observed above for an unheated air knife (Figure 2) up to and just past the retrograde solubility temperature. Comparing 80 to 60 °C, accelerating nucleation in the retrograde solubility regime had the desired effect of reducing the average size of the voids. However, as the temperature increased beyond 80 °C, larger microstructures and larger voids appeared, which can be attributed to unconstrained vertical growth of crystals and favored Ostwald ripening.^32^

To further understand the different processing conditions and their correlation with the drying rate and nucleation of perovskite, our team recently reported a quasi-two-dimensional (2D) growth model that utilizes the mass transfer Biot number.^29^ For an evaporating thin film, the traditional mass transfer Biot number (*Bi*) can be described as the ratio of diffusional mass transfer resistance within the film to evaporative mass transfer resistance at the surface of the film.^33,34^ Traditionally, diffusion and evaporation are defined in the same direction, both orthogonal to the surface of the film. However, because the primary growth direction governing film coverage is in the horizontal direction, we use the aspect ratio (Λ, where *b* is the half-distance between nuclei and *h*_0_ is approximated by the average of peak heights of microstructures) to recast the Biot number based on the characteristic horizontal diffusion between nuclei. Equation 1 defines the modified Biot number (Bi*)
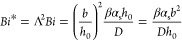
1where β, α_s_, and *D* represent the mass transfer coefficient, solvent liquid–vapor volume ratio, and diffusion coefficient, respectively. For Bi* ≪ 1, crystal growth is not diffusion-limited; instead, lateral diffusion is fast, and the evaporative mass transfer rate is slow, leading to taller, disconnected crystalline domains. For Bi* ≫1, evaporative mass transfer rates are fast, leading to significant vertical confinement as the wet film thickness quickly decreases, and the crystal growth becomes primarily horizontal. Bi* > 1 is desirable to achieve a densely interconnected film. The estimated Bi* values for processing with pure DMF or DMSO are in the range of 1–3 for temperatures of ∼100 °C. State-of-the-art devices are often made using solvent blends, where a large Bi* is achieved by combining a high vapor pressure solvent (such as DMF) to enhance evaporation and increase *Bi*, as discussed in our prior work,^29^ with a strongly complexing solvent that can moderate nucleation (such as DMSO),^35^ thereby increasing Λ. Details of the derived model and simulated film profiles are provided by Starger et al.,^29^ and Bi* calculations are summarized in the Supporting Information.

**Figure 5:**
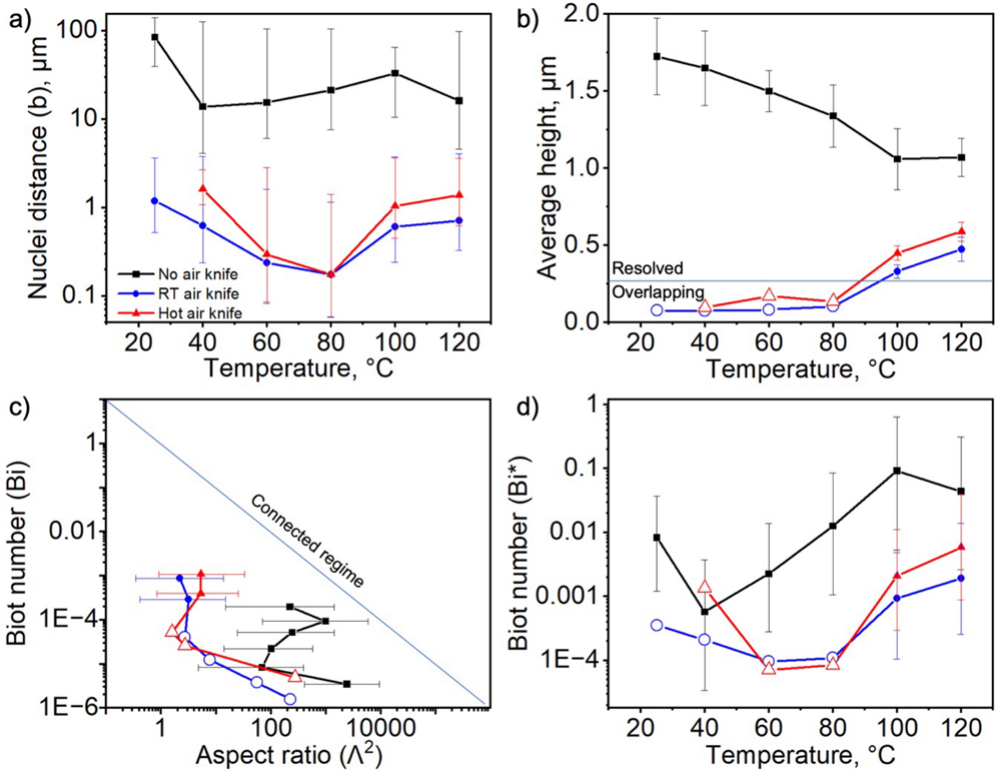
Crystallization parameters determined from image analysis of dry films. (a) Distance between microstructures. (b) Microstructure height of perovskite films. (c) Process map of *Bi* and the aspect ratio for a range of processing conditions. (d) Estimated Bi* for these processing conditions. Error bars in panel *a* represent the range of values measured in the distribution, with outliers removed, and those in panel *b* represent the standard deviation of the heights. Open symbols are shown for quantities based on height measurements, where the individual domains are not well-resolved. Details on the determination of Λ, *Bi*, and Bi* are in the Supporting Information.

Figure 5a shows the distance between microstructures (surviving nuclei) estimated from optical microscopy or AFM images. Statistics and details regarding image processing are shown in Figures S8–S10 of the Supporting Information. The AFM images were also processed to estimate the average domain height, as shown in Figure 5b; however, owing to challenges in determining this quantity when the domains are small and frequently overlapping, only a “lower limit” is presented in cases of an air knife with a temperature below 100 °C (indicated by open symbols on the plot). Open symbols are also used for Λ and Bi* in such cases because those parameters depend upon *h*_0_. The obtained values of *Bi* are plotted against Λ^2^ on log–log axes to evaluate and map the trends (Figure 5c). Figure 5d illustrates the trend of the estimated Bi* values for all investigated processing conditions, which consistently exhibit significantly lower values by 2–3 orders of magnitude compared to solvents commonly used to create more uniform films.

Rapid drying of films by increasing the temperature or forced convection resulted in increased *Bi* but not always increased Bi*. Trends in Bi* are hard to predict *a priori*: while increasing the temperature and adding convection are both expected to enhance solvent evaporation, they can also influence nucleation and Ostwald ripening rates, thereby changing *b*. The influence of these parameters on *b* in other solvents cannot be generalized. As a result of the quadratic dependence of Bi* upon *b* and the fact that *h*_0_ is limited to within an order of magnitude of the average final film thickness, small *b* values necessarily result in extremely small Bi* values. Contrary to our initial objective, Bi* was made smaller by adding convection, illustrating that we have moved farther from the condition in which solvent-confined crystallization facilitates connectivity between domains. The highest uniformity observed herein was instead achieved at the expense of the formation of large domains: rather than orderly crystal growth, the morphology of the films in the 60–80 °C range appears to be controlled by dense nucleation. However, films with overly small domains may well be salvaged by post-deposition annealing to consolidate domains,^36^ a step that was not performed here. Indeed, in continuous coating examples for which the addition of convection was observed to enhance final uniformity, post-deposition annealing was performed.^4,37,38^

For processing temperatures above 80 °C, the number of crystallites observed in the final dried film decreases with increasing temperature (as seen by AFM in Figure 4b) without the desired enhancement in uniformity. In terms of the void size and film roughness, these are the worst films. This would appear to run contrary to both the expectation of denser crystallites as a result of the retrograde-solubility-driven higher nucleation rate and the prediction of greater coverage with greater *b*. However, neither of these expectations takes into account the acceleration of Ostwald ripening with the temperature. Ostwald ripening is so robust at 100 °C that it can be easily discerned in real-time microscopy videos of the evaporation of droplets of precursor solution (see still images in Figure S7 of the Supporting Information). If Ostwald ripening is rampant, the approach herein of estimating nucleation density from the density of domains in the final dried film provides only an upper limit on the value of *b* and, hence, an upper limit on the value of Bi*. The central conclusion of this work is that Bi* remains critically below 1, even with convection, which is increasingly valid if *b* is overestimated. Development of *in situ* rather than post-mortem methods of determining *b* would be valuable for cases with significantly larger Bi* values than those explored here.

## Conclusion

In conclusion, an analysis of the microstructure, coverage, and film quality of MAPbI_3_ deposited under conditions of retrograde solubility is presented and the concept of the modified mass transfer Biot number (Bi*) is applied. The ability of retrograde solubility to enhance the nucleation rate was evaluated. To independently tune the drying and nucleation rates, an air knife and heated air knife were employed, along with variations in the stage temperature. The introduction of either form of forced convection significantly improved film coverage by enhancing nucleation density, particularly up to 80 °C. The slow evaporation of GBL nonetheless inhibits the desired pinhole-free morphology, because each nucleus grows to a great extent before the evaporation of solvent has a chance to reduce the wet film height and constrain the vertical growth. This disconnected growth regime is reflected by Bi* being roughly 2–3 orders of magnitude less than what is needed for uniform films (Bi* > 1). Attempting to increase Bi* through independent control of the temperature and convection was shown to be limited by the strong coupling between the temperature, drying rate, and nucleation, resulting in generally little to no overall change in Bi*. Increasing the temperature further to exploit the concerted effects of rapid evaporation and retrograde solubility still cannot overcome this limitation, because Ostwald ripening is rampant at such high temperatures and the nuclei are quickly consumed. This experimental and quantitative assessment approach has provided a detailed understanding of the interplay among perovskite material processing, morphology, and solvent parameters and points out the significant impediments to uniformity for any solvent with an excessively high boiling point. The approach can be extended to identify suitable solvent systems for the processing of perovskite materials in large-area optoelectronic applications.

## Supporting Information Available

Photograph of the slot-die coater with the air knife setup, AFM images, microscopic images, XRD data, statistics from image processing, and details of Biot number calculations (PDF)
