# Supplementary material for: Retrograde Solubility of Methylammonium Lead Iodide in γ-Butyrolactone Does Not Enhance the Uniformity of Continuously Coated Films
Source: Langmuir. 2024 Apr 18;40(17):8836–42. doi: 10.1021/acs.langmuir.3c03979 (PMC11197085; doi:10.1021/acs.langmuir.3c03979)
Supplement: Supplementary file 1 — la3c03979_si_001.pdf [file la3c03979_si_001.pdf]

Supporting Information

Retrograde Solubility of Methylammonium Lead Iodide in Gamma-Butyrolactone Does Not Enhance the Uniformity of Continuously Coated Films

Maimur Hossain[1], Jesse Starger[1], Jesse J. Efymow[1], Ryan F. Barrett[1], Jacob S. Bolduc[2], Nicolas J. Alvarez[1], Richard A. Cairncross[1], Aaron T. Fafarman[1], Jason B. Baxter[1*]*

[1]Department of Chemical and Biological Engineering, Drexel University, Philadelphia, PA 19104, USA

[2]Department of Materials Science and Engineering, Drexel University, Philadelphia, PA 19104, USA

*Address correspondence to: jbaxter@drexel.edu, atf37@drexel.edu

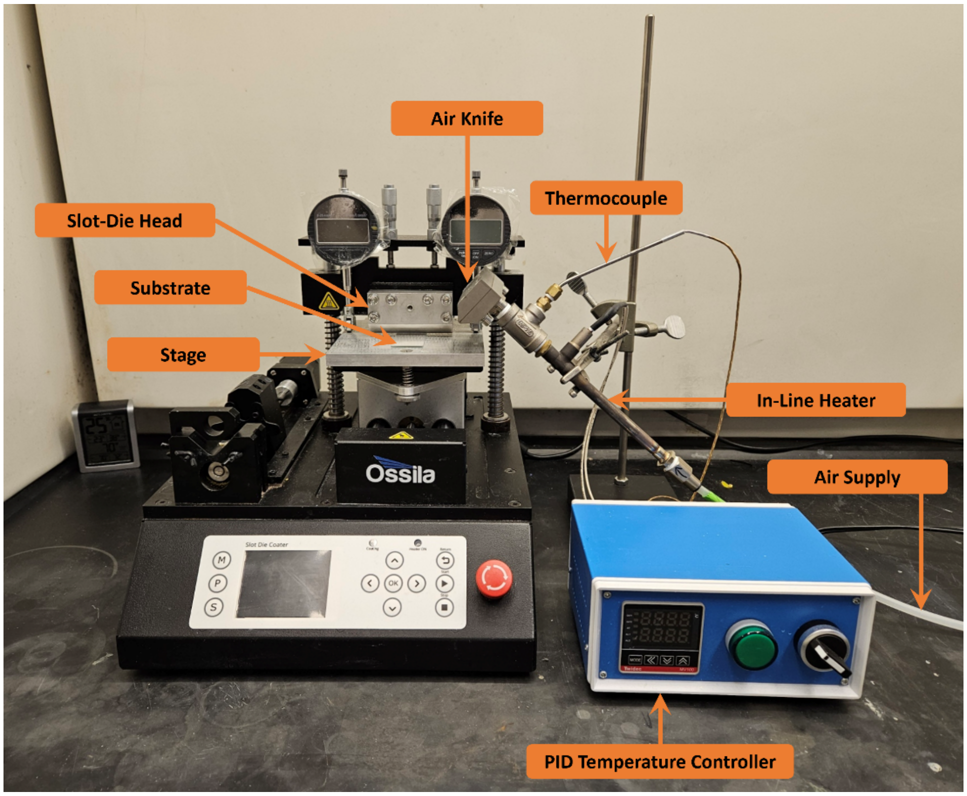

Figure S1. Labeled photograph of slot-die coater and heated air knife setup.

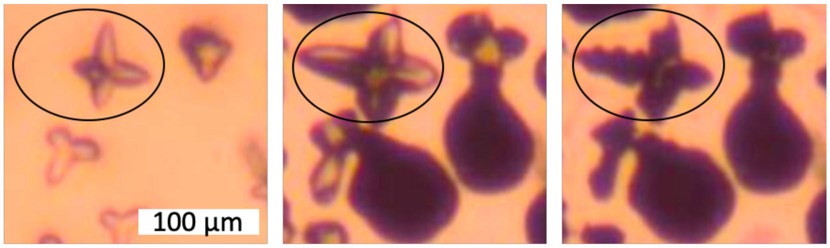

Figure S2. Series of three still images taken from a video of initial crystallization and subsequent drying and phase transition of a droplet of MAPI in GBL held at 60 °C. The gross star-shape morphology of the transparent, putative $MAPbI_3$:GBL adduct crystal phase is retained in the black, apparently polycrystalline final solid, as is most readily seen for the circled microcrystal in the upper left. During drying, after a period in which only transparent microcrystals are observed, amorphous black solids begin to be nucleated off of the first crystals.

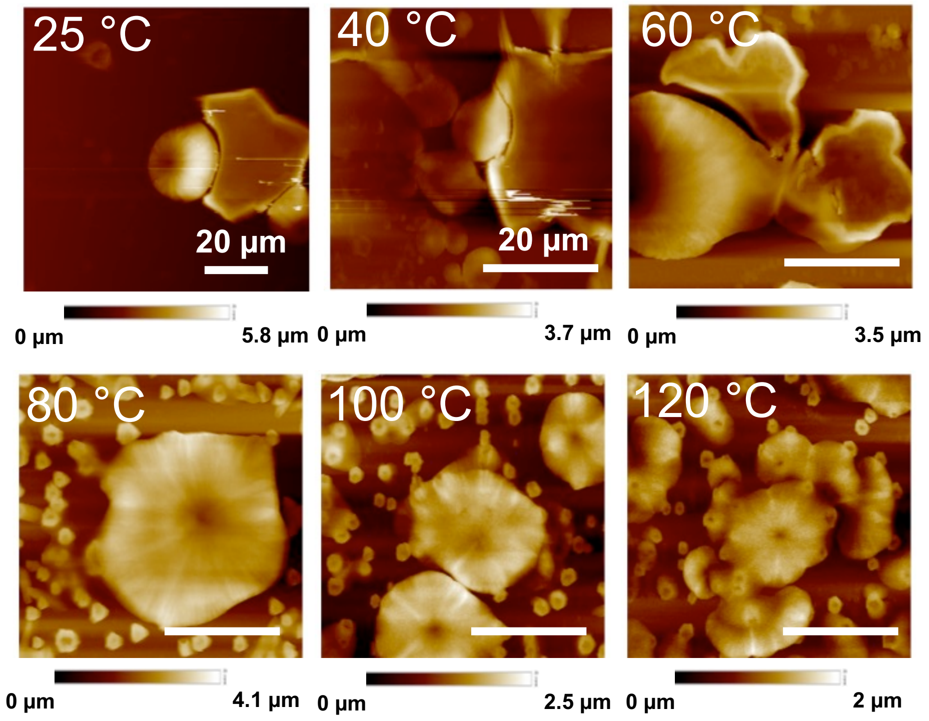

Figure S3. AFM images of perovskite films coated with varied stage temperature without air knife. All scale bars are 20 μm (only 25 °C image is of different magnification).

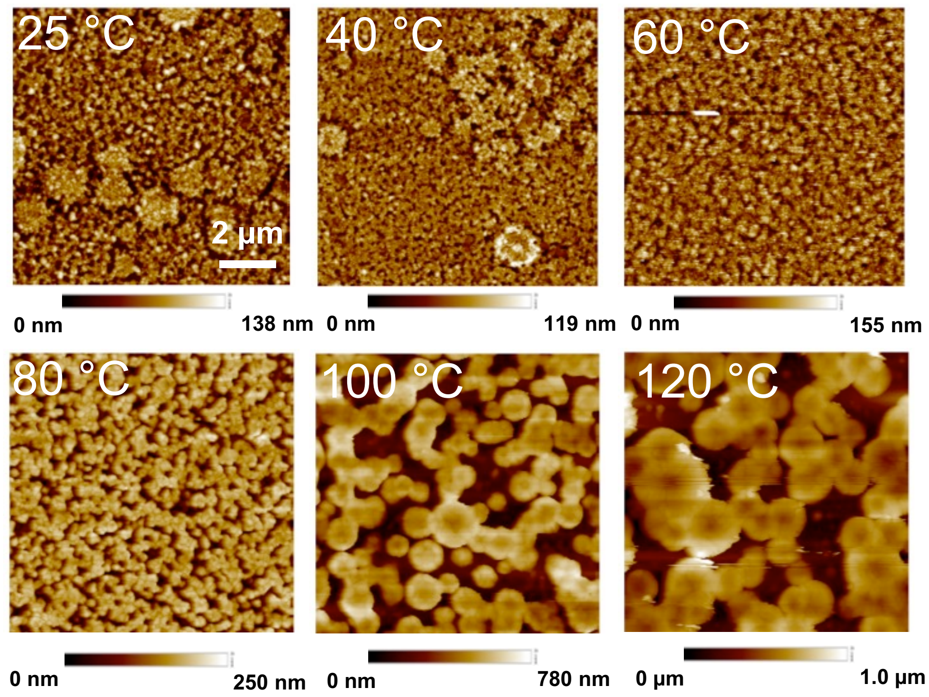

Figure S4. AFM images (scale bar of 2 μm applies to all images) of perovskite film coated using varied stage temperature and with air knife.

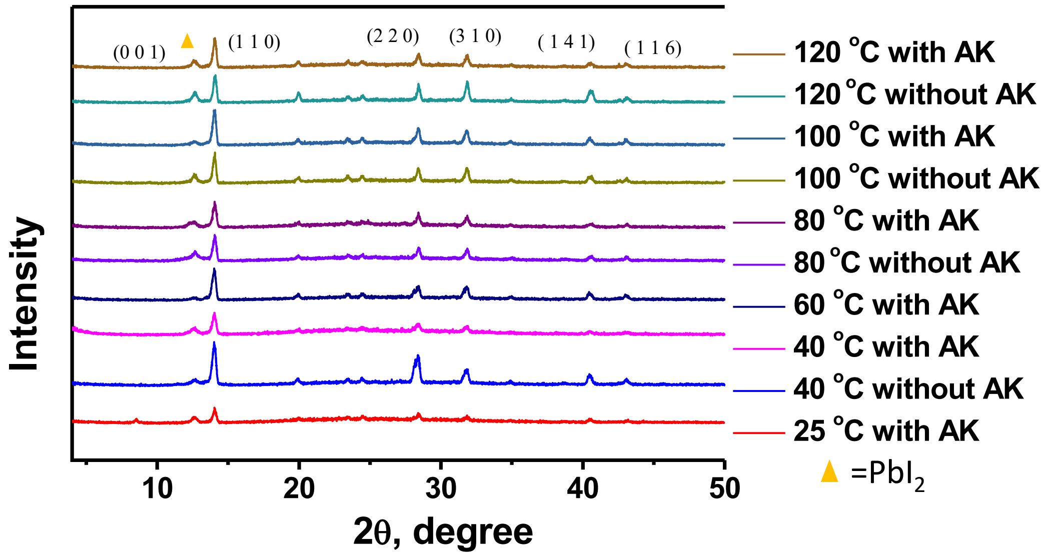

Figure S5. XRD pattern of perovskite films processed at different temperatures and with or without air knife.

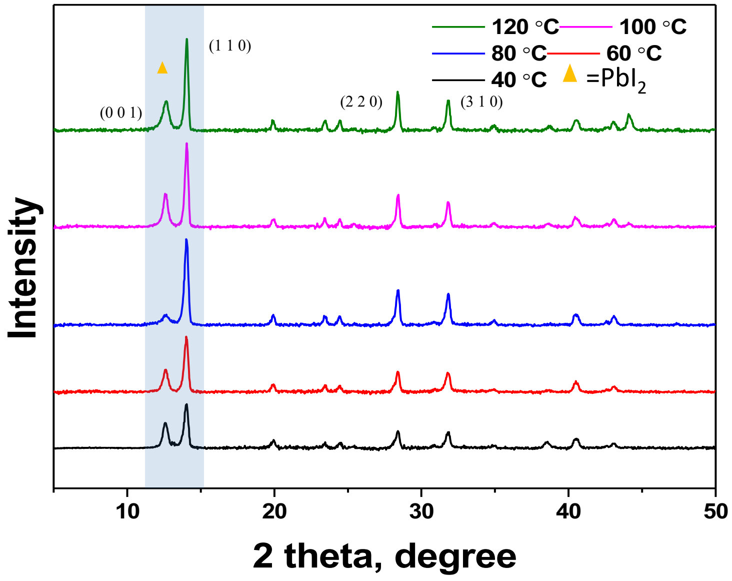

Figure S6. XRD pattern of perovskite film coated with hot air knife at different temperatures. All the films had three major intense peaks of (1 1 0), (2 2 0) and (3 1 0) perovskite planes at 14.1°, 28.4° and 31.8° along with smaller PbI_2 (001) peak at 12.7°.

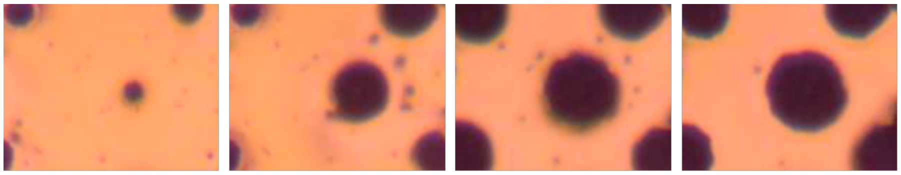

Figure S7. Series of four still images taken from a video of initial crystallization and subsequent drying of a droplet of MAPI in GBL held at 100°C. Initially, a single microcrystal is clearly resolved in the central region of the image. It grows and several smaller crystallites are observed in its immediate vicinity. The same crystallites appear smaller in a subsequent image and finally all but one have disappeared in the final image as the central microcrystal continues to grow, presumably at the expense of the smaller ones.

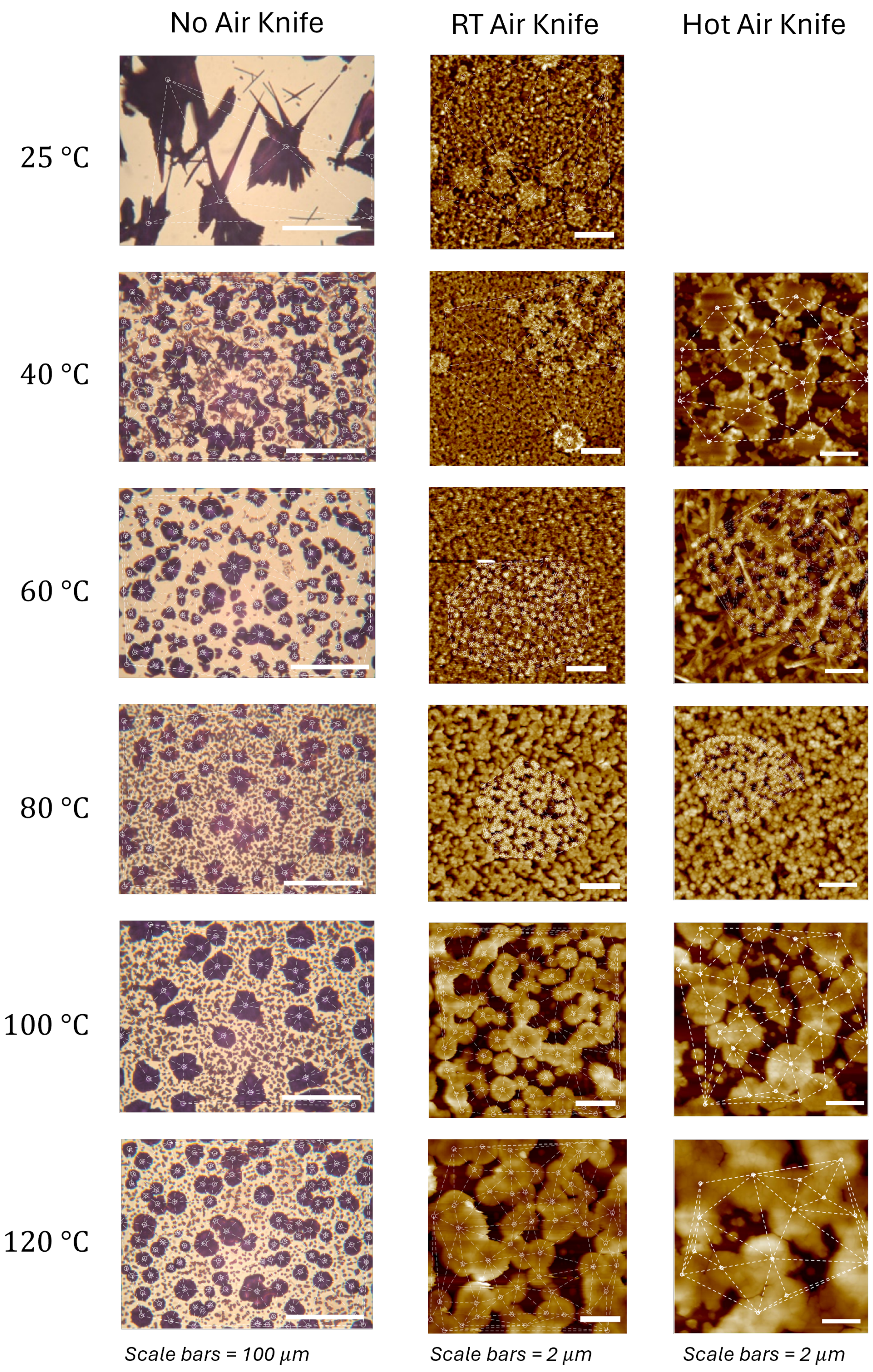

Figure S8. Brightfield microscope (No air knife) and AFM (RT Air Knife, Hot Air Knife) images showing mapping of crystal domain centers (open circles) and triangulated nearest neighbor distances (dashed lines) between centers. Domain centers were found using ImageJ, and Delaunay triangulation was performed in MATLAB.

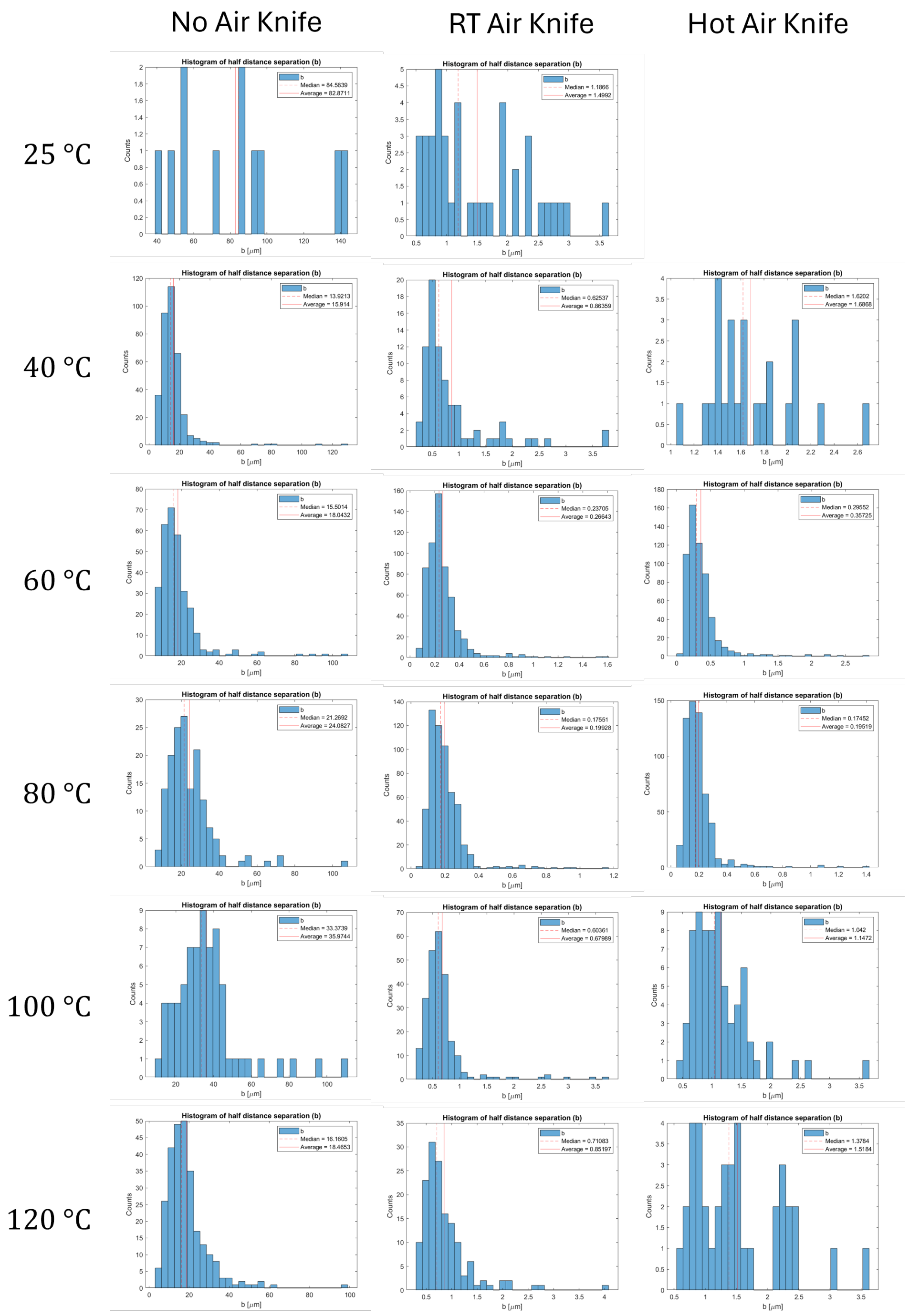

Figure S9. Histograms of all half-distance separation b values measured from triangulated nearest neighbor distances measured. Population median and averages are reported and overlaid as dashed red lines and solid red lines, respectively.

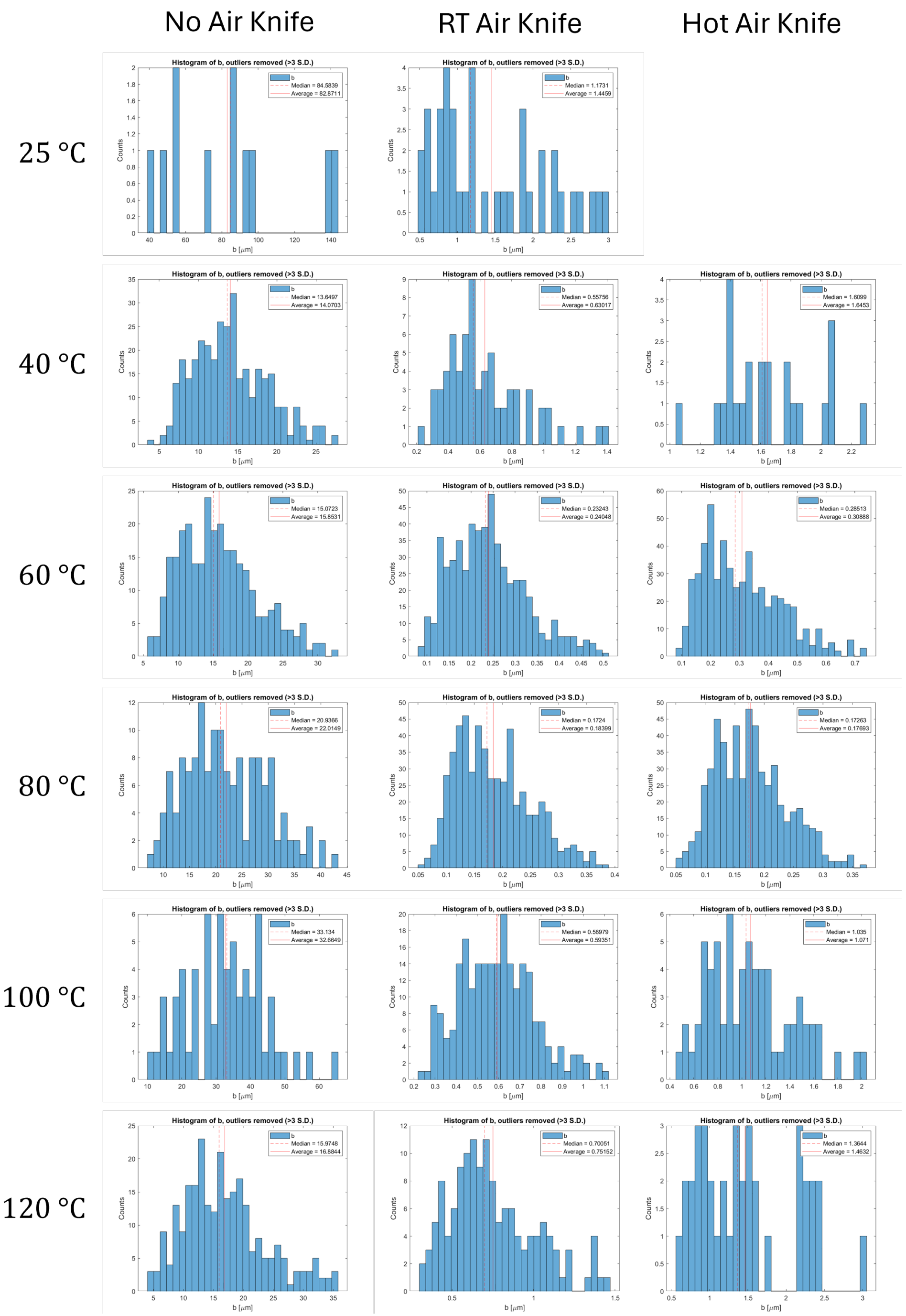

Figure S10. Histograms of half-distance separation *b* values with outliers greater than three standard deviations from the median removed to exclude edge measurements (unrealistically large b values). Population median and averages are reported and overlaid as dashed red lines and solid red lines, respectively.

Biot number calculation:

The solvent expansion coefficient, α_s, is defined as

$$\alpha_s = \frac{p_{vap}}{RT}\bar{V}_S,$$

where p_{vap} is the solvent vapor pressure, R is the gas constant, T is temperature, and $\bar{V}_S$ is the solvent liquid molar volume. The estimated distances between nuclei, thickness of dry film, and $Bi*$ are presented in Figure 5 for different processing conditions. In the absence of an air knife, the mass transfer coefficient (β) for film deposition was 10^{-3} m/s as determined by Ternes *et al;* however, when an air knife or hot air knife was employed, the β value increased to 10^{-2} m/s.[S1]

Error bars shown in Fig. 5 represent the ranges of possible $Bi, \Lambda,$ and Bi^* calculated based on the following relationships (where h_{SD} represents one standard deviation from the mean height):

$$Bi_{avg} = \frac{\beta h_{avg}}{D}\alpha_s$$

$$Bi_{min} = \frac{\beta h_{min}}{D}\alpha_s, \qquad \text{where } h_{min} = h_{avg} - h_{SD}$$

$$Bi_{max} = \frac{\beta h_{max}}{D}\alpha_s, \qquad \text{where } h_{max} = h_{avg} + h_{SD}$$

$$\Lambda^2_{median} = \left(\frac{b_{median}}{h_{avg}}\right)^2$$

$$\Lambda^2_{min} = \left(\frac{b_{min}}{h_{max}}\right)^2$$

$$\Lambda^2_{max} = \left(\frac{b_{max}}{h_{min}}\right)^2$$

$$Bi^*_{median} = Bi_{avg}\,\Lambda^2_{median}$$

$$Bi^*_{min} = Bi_{min}\,\Lambda^2_{min}$$

$$Bi^*_{max} = Bi_{max}\,\Lambda^2_{max}$$

Reference

S1. Ternes, S., Börnhorst, T., Schwenzer, J. A., Hossain, I. M., Abzieher, T., Mehlmann, W., Lemmer, U., Scharfer, P., Schabel, W., Richards, B. S., Paetzold, U. W., Drying Dynamics of Solution-Processed Perovskite Thin-Film Photovoltaics: In Situ Characterization, Modeling, and Process Control. *Adv. Energy Mater.* 2019, 9, 1901581. https://doi.org/10.1002/aenm.201901581.
